# Sexual Dimorphism of miRNAs Secreted by Bovine *In vitro*-produced Embryos

**DOI:** 10.3389/fgene.2017.00039

**Published:** 2017-04-04

**Authors:** Nicole Gross, Jenna Kropp, Hasan Khatib

**Affiliations:** Department of Animal Sciences, University of Wisconsin, MadisonWI, USA

**Keywords:** microRNA, sexual dimorphism, embryo, culture media, bovine

## Abstract

Sexual dimorphism of bovine blastocysts has previously been observed through differences in development, cell death, metabolism, telomere length, DNA methylation, and transcriptomics. However, dimorphism in the secretion of miRNAs to culture media has not yet been evaluated. The objectives of this study were to determine if sex-specific blastocyst miRNA secretion occurs and to further investigate the role these miRNAs may have in the interaction between a blastocyst and the maternal environment. *In vitro* embryo culture was performed and media from male and female blastocysts was collected into sex-specific pools. Profiling of 68 miRNAs revealed a total of eight miRNAs that were differentially expressed between female and male-conditioned media. Validation by qPCR confirmed higher expression of miR-22 (*P* < 0.05), miR-122 (*P* < 0.05), and miR-320a (*P* < 0.05) in female media for three additional biological replicates. To examine the potential roles of secreted miRNAs to the media in communication with the maternal environment, miR-22, miR-122, and miR-320a were each supplemented to four replicates of primary bovine endometrial epithelial cell culture. Uptake of miR-122 (*P* < 0.05) and miR-320a (*P* < 0.05) was detected, and a trend of uptake was detected for miR-22 (*P* > 0.05). Further, expression of the progesterone receptor transcript, a predicted target of all three miRNAs, was found to be upregulated in the cells following supplementation of miR-122 (*P* < 0.05) and miR-320a (*P* < 0.05), and a trend upregulation of the transcript was observed following miR-22 (*P >* 0.05) supplementation. This work demonstrates that male and female conceptuses are able to differentially secrete miRNAs at the blastocyst stage and that these miRNAs have the ability to induce a transcriptomic response when applied to maternal cells. This knowledge builds on the known dimorphic differences in conceptuses at the blastocyst stage and demonstrates a role for blastocyst-secreted miRNAs in cell–cell communication.

## Introduction

Mammalian embryos exhibit sexual dimorphism in development, genetics, and epigenetics (Laguna-Barraza et al., 2013). *In vitro* production systems employed to generate bovine embryos, have reported that male embryos develop faster (Avery et al., 1992) and have increased blastocyst rates (Camargo et al., 2010; Ghys et al., 2016) as well as higher total cell numbers (Xu et al., 1992; Ghys et al., 2016; Oliveira et al., 2016) compared to female embryos. On day 7 of development, *in vitro*-produced female bovine blastocysts show a higher incidence of cell apoptosis than male blastocysts (Ghys et al., 2016), with the earliest detection of this effect on day 6 (Oliveira et al., 2016). Dimorphism in terms of metabolic strategy is also a distinct; this is demonstrated by the differential uptake of amino acids between male and female embryos (Sturmey et al., 2010), and was shown in a study by Green et al. (2016) in which alteration of glucose availability in culture medium induced a stronger skew in sex ratio of bovine blastocysts.

When cultured under the same conditions, embryos exhibit sexual dimorphism in up to one-third of actively expressed genes (Bermejo-Álvarez et al., 2010). Further, when exposed to adverse culture conditions, male and female conceptuses exhibit differentially altered transcriptomes (Heras et al., 2016). Additionally, mitochondrial distribution, telomere length, and DNA methylation have shown sexual dimorphism in bovine embryos (Bermejo-Álvarez et al., 2008). Though differences have been detected between male and female blastocysts, a better understanding of the role these changes play in communication to the maternal environment is warranted.

To fully understand successful pregnancy, it is important to elucidate the signaling mechanisms, or dialog, between an embryo and the mother. A potential signaling mechanism involved in regulation of embryo development is the secretion of miRNAs into the extracellular environment. miRNAs are a family of short, single-stranded, non-coding RNAs that are approximately 19–25 nucleotides in length. These small molecules modulate sequence-specific mRNA transcription, leading to regulation of gene expression (Wahid et al., 2010). Sexually divergent miRNA expression may contribute to observed differences between male and female embryos. As shown on the miRbase archive^1^ (release 21; Kozomara and Griffiths-Jones, 2014), the Bos taurus Y chromosome has no known miRNAs, whereas the X chromosome encodes 61 known miRNAs. miRNAs can be secreted out of a cell through extracellular vesicles, via apoptotic bodies, or by being bound to AGO proteins (Turchinovich et al., 2013). In cattle, the presence of circulating miRNAs has been reported in bodily fluids such as milk (Sun et al., 2015), blood, and follicular fluid (Noferesti et al., 2015) and in the *in vitro* culture media of blastocysts (Kropp et al., 2014; Kropp and Khatib, 2015). miRNAs have shown involvement in a variety of biological processes including organismal development, cell proliferation, cell death, hematopoiesis, and immunity (Wahid et al., 2010). Evidence that miRNAs modulate signaling networks, such as the purinergic network — an extracellular network mediated by nucleosides such as adenosine and ATP (Ferrari et al., 2016) — supports the potential of embryo-secreted miRNAs to serve as important pathway regulators.

Embryo-secreted miRNAs have demonstrated potential to affect endometrial transcriptomes. For example, miR-30b transfected into human endometrial epithelial cells induced transcriptomic changes in the cells (Ye et al., 2015). miRNA-661 has been shown to decrease maternal protein expression, and correlate with implantation outcome (Cuman et al., 2015). The ability of these miRNAs to alter uterine cell transcriptomes and protein expression further warrants a more comprehensive investigation of embryonic miRNAs as signaling molecules to the mother. However, it is not yet known whether miRNAs present in the culture medium of *in vitro*-produced embryos differ between male and female embryos. It is unknown if these miRNAs serve a function in the dialog between the embryo and the mother. We hypothesized that sexual dimorphism exists in the profiles of miRNAs secreted by blastocysts, and that these miRNAs serve as a signaling mechanism for male and female conceptuses to alter the gene expression profile in maternal endometrial cells.

## Materials and Methods

### Ethics Statement

This study is exempt from approval of the institutional and national requirements of Animal Care and Use Committee because cows used for *in vitro* fertilization were not handled at the University of Wisconsin facilities. Ovaries used for embryo production were purchased from Applied Reproductive Technology, LLC (Monona, WI, USA), and permission was granted by the company to perform *in vitro* fertilization using ovaries.

### Experiment I: miRNA Profiling in Media

#### *In vitro* Embryo and Media Production

Embryos and media were procured as described by Kropp and Khatib (2015). Ovaries were obtained from Applied Reproductive Technology, LLC (Monona, WI, USA), and follicles were aspirated to recover oocytes. Oocytes were washed in Vigro TL-Hepes (Bioniche, Pullman, WA, USA) supplemented with 3% bovine serum albumin, sodium pyruvate, and gentamicin and placed in cohorts of 10 oocytes per 50 μl drop of maturation media for 24 h. Maturation media consisted of M-199 media supplemented with gonadotropins (FSH and LH), estradiol, sodium pyruvate, 10% fetal bovine serum and gentamicin. Following 24 h of maturation, cumulus-oocyte complexes were washed in Vigro TL-Hepes (Bioniche) and transferred in cohorts of 10 into a 44 μL drop of fertilization media supplemented with fatty acid-free bovine serum albumin (FAF-BSA), sodium pyruvate, and gentamicin. Sperm was prepared using a Percoll discontinuous gradient as described by Parrish et al. (1995), where the final concentration was adjusted to 1 million sperm per mL and 2 μL was added per drop. Additionally, 2 μl each of penicillamine-hypotaurine-epinephrine and heparin were added to each fertilization drop.

Gametes were co-cultured with sperm for 20 h at which point presumptive zygotes were stripped of their cumulus cells and washed in supplemented Vigro TL-Hepes (Bioniche). The presumptive zygotes were placed 25 per drop into a 50 μL drop of CR_1aa_ culture media (Rosenkrans et al., 1993; Sagirkaya et al., 2006) supplemented with FAF-BSA, sodium pyruvate, amino acids, and gentamicin. Embryos were then cultured until day 5, whereupon they were morphologically assessed for characteristics of the morula stage as described by Bó and Mapletoft (2013). Morulae were selected based on the appearance of a coalesced/compacted inner cell mass that consumed 60–70% of the perivitelline space. Those deemed as morula stage embryos were washed and placed individually into a 50 μL drop of CR_1aa_ medium lacking FAF-BSA supplementation.

On day 8 of development, individually cultured embryos were morphologically assessed for characteristics of the blastocyst stage of development. Embryos which developed a blastocoel cavity and demonstrated a distinct inner cell mass and outer trophectoderm were deemed blastocysts, whereas those which failed to develop to the blastocyst stage were deemed degenerate embryos. Blastocysts that were at the mid-to-expanded blastocyst stage and quality grades 1 and 2 (Bó and Mapletoft, 2013), as well as each embryo’s respective conditioned culture media, were individually collected. Blastocysts were stored in 10 μL TE buffer and media was preserved in aliquots. Both embryos and media were stored at -20°C for subsequent procedures. Two different sires were used for embryo generation.

#### Sex Determination of Embryos by Nested PCR

Individual embryos were genotyped for sex determination as described by Kirkpatrick and Monson (1993). In brief, PCR was performed using the GoTaq^®^ DNA Polymerase system (Promega Corporation, Madison, WI, USA). Reagents [5X Green GoTaq^®^ Reaction Buffer, dNTPs, magnesium chloride and primers (Supplementary Table 1)], as well as proteinase K, were added to a PCR reaction containing one blastocyst embryo in 10 μl of TE buffer. To lyse each embryo, PCR reactions were incubated at the following temperatures: 50°C for 30 min, 95°C for 10 min, -20°C for 5 min, 95°C for 10 min, and -20°C for 5 min. Taq DNA polymerase was then added to each well and the first PCR was carried out with primers zfx/zfy (Aasen and Medrano, 1990; Kirkpatrick and Monson, 1993) under the following conditions: 95°C for 5 min, followed by 30 cycles of 95°C for 60 s, 55°C for 45 s, and 72°C for 60 s, and a final extension step of 72°C for 7 min. A nested PCR was then performed with primers specific to the X (zfx) and Y (zfy) chromosomes using the initial PCR product as template. The second PCR was cycled under the same conditions as the initial PCR. Embryo sex was confirmed by gel electrophoresis, where a single band was visualized for a female (X chromosome product of 246 base pairs) or two bands were visualized for a male (X chromosome band plus a Y chromosome product of 167 base pairs). A total of three IVF replicates were carried out to procure media samples derived from female and male embryos in which 102 blastocyst embryos were collected. Of the blastocysts collected, 57 blastocyst embryos were successfully genotyped, where 30 were male and 27 were female (Supplementary Table 2).

#### RNA Extraction from Culture Media and miRNA Profiling

For miRNA differential expression analysis between female and male embryos, a total of three pools of media were generated each for males (each pool consisted of media from 10 embryos) and females (each pool consisted of media from 9 embryos). Embryo pools were determined following IVF completion. Each pool included media from a unique set of individual embryos. Pools were designed to contain media of embryos from all three IVF runs, representing both sires equivalently across pools. RNA was extracted from each pool using a miRNeasy Serum/Plasma kit (Qiagen, Germantown, MD, USA). A total of three extractions were carried out per pool, with an initial input volume of 120–140 μL media sample, consisting of an equal volume of eluent from individual embryos. miRNA profiling was performed using the Firefly^®^ Circulating miRNA Assay Immunology Panel (ABCAM, Cambridge, MA, USA). The immunology panel includes 68 immune response-related miRNAs chosen by ABCAM based on known functions related to immune response and differential regulation in plasma or serum. We hypothesized that these miRNAs could play a role in the immune response of the mother to the developing embryo. Complementary oligonucleotides which encode hydrogel microparticles were hybridized to selected miRNAs. The oligonucleotide adapter served as a universal PCR priming site, allowing for fluorescent amplification of the target. Amplified products were then re-hybridized to original oligonucleotide particles, and an EMD Millipore Guava 8HT Flow Cytometer (Merck, Darmstadt, Germany) was used to quantify the hybridization. Measurements were performed in triplicates.

Statistical analysis of differentially expressed miRNAs was performed using the Firefly^®^ Analysis Workbench Software (ABCAM) in which miRNA expression levels of male and female embryos were compared to each other. An unpaired *t*-test was used to determine significant differential expression (*P* < 0.05) of miRNAs between male and female embryo groups.

#### Validation of Differentially Expressed miRNAs Using Quantitative Real-time PCR (qRT-PCR)

For the validation of differentially expressed miRNAs detected by Firefly^®^ particle technology, three miRNAs (miR-22, miR-122, and miR-320a) were chosen for further analysis by qRT-PCR. A total of five additional IVF replicates were performed to generate 146 embryos derived from one sire and different dams. Fertilization, embryo grading, media collection, and embryo genotyping were performed as described above. A total of 101 blastocysts embryos were successfully genotyped for validation, resulting in 51 male and 50 female blastocysts (Supplementary Table 2). Male and female pools of media, with volumes of 40–90 μL, were created and RNA was extracted as described above. The three male pools were derived from 17 embryos each, while three female pools were derived from 17, 16, and 16 embryos. Total RNA was reverse-transcribed using a miScript II RT kit (Qiagen) with HiSpec Buffer following the manufacturer’s instructions. The qRT-PCR method was used to determine relative fold difference in miRNA expression between male and female pools. A miR-39 spike-in control (Qiagen) was added at the time of extraction to serve as an internal control. The miScript SYBR Green Kit (Qiagen), and specific primers (Supplementary Table 3) corresponding to each mature miRNA sequence (Qiagen) were used. Cycling conditions were carried out in a Bio-Rad iCycler real-time PCR machine as follows: 95°C for 15 min followed by 40 cycles of 94°C for 15 s, 55°C for 30 s, and 70°C for 30 s. C_T_ values > 33 were considered beyond the threshold for detection. The 2^-ΔΔC_T_^ method by Livak and Schmittgen (2001) was used to determine mean fold change in miRNA gene expression, where ΔΔC_T_ = (C_T,Target miRNA_ - C_T,miR-39_)_female media_ - (C_T,Target miRNA_ - C_T,miR-39_)_male media_. The ΔC_T_ values were evaluated for significance using an unpaired *t*-test for each miRNA. The TargetScan software^2^ (Lewis et al., 2005) was used to determine candidate target genes of validated miRNAs. Each miRNA was searched by name, under both cow and human for species selection. Top predicted targets were viewed. Those which were targeted in both cow and human, and additionally had known roles in embryo development were manually identified.

### Experiment II: Uptake of miRNAs into Bovine Endometrial Cells

#### Primary Cell Culture of Bovine Endometrial Cells

To investigate the potential of validated miRNAs as signaling molecules to maternal tissues, a primary cell culture system was implemented. Bovine endometrial epithelial cells (BEECs) (Cell Applications Inc., San Diego, CA, USA) were cultured for 6–8 passages and seeded in a 96-well culture plate at 7000 cells per cm^2^ per manufacturer instruction. Cells were cultured in Bovine Endometrial Epithelial Cell Growth Medium (Cell Applications Inc.) and passaged using Hank’s Balanced Salt Solution, Trypsin/EDTA and Trypsin Inhibitor (Cell Applications Inc.), as directed.

#### miRNA Supplementation and Collection of Primary Endometrial Epithelial Cells

Synthetic mimics for the miRNAs miR-22, miR-122, and miR-320a (Qiagen) — specific to those found to be differentially expressed between male and female embryos — were supplemented to BEECs to assess whether these miRNAs modulate maternal gene expression. At 24 h post-passage of cells, 50 nM mimic miRNA was added to a well of a 96-well plate containing BEECs. Notably, no transfection reagent was used in order to more closely simulate an *in vivo* setting. A control of non-treated cells was simultaneously cultured. A total of four biological replicates were produced for each miRNA mimic treatment. After 24 h of co-culture with the miRNA mimic, the medium was aspirated from each well, cells were washed twice in 200 μL PBS, and lifted with trypsin. Cells were pelleted at 300 g for 5 min, and the excess supernatant was aspirated from each tube and discarded.

To evaluate the effect of miRNAs on gene expression in the endometrial epithelial cells, an additional experiment was performed, in which cells were treated with 1 μL of Lipofectamine 2000 (Thermo Fisher Scientific, Madison, WI, USA) in conjunction with 50 nM of miRNA and Opti-MEM medium, as specified by the manufacturer. A control of lipofectamine-only treated cells was cultured simultaneously.

#### Primary Endometrial Epithelial Cell miRNA and mRNA Extraction and Quantification

Total RNA was extracted from the BEECs using a MiRNeasy Mini Kit (Qiagen) to assess miRNA uptake by the maternal cells and expression of genes targeted by supplemented miRNAs, respectively. Reverse transcription and quantification of uptake of miRNAs in cells were performed as described for media. Reverse transcription of mRNA was carried out using an iScript cDNA kit (Bio-Rad, Hercules, CA, USA) and qRT-PCR to evaluate the expression of candidate mRNA targets was performed using iTaq Universal SYBR Green Supermix (Bio-Rad). The β-actin gene was selected as an internal control according to its stability across samples in comparison to GAPDH. Intron-spanning primers were designed for each target mRNA to avoid amplification of genomic DNA (Supplementary Table 4). The CFX Connect Real-Time PCR Detection System was used for mRNA quantification with the following cycling conditions: 95°C for 30 s, followed by 40 cycles of 95°C for 5 s, and 60°C for 30 s. Gene expression analysis was performed using the 2^-ΔΔC_T_^ method (Livak and Schmittgen, 2001), with a control group of non-treated cells. To assess the effects of supplementation and mRNA expression changes, biological replicates were standardized as previously described (Willems et al., 2008), maximally reducing inter-sample variation between cells. Following log transformation, mean centering, and autoscaling, an unpaired *t*-test was performed to assess significance (*P* < 0.05).

## Results

### Embryo Genotyping and miRNA Profiling

**FIGURE 1:**
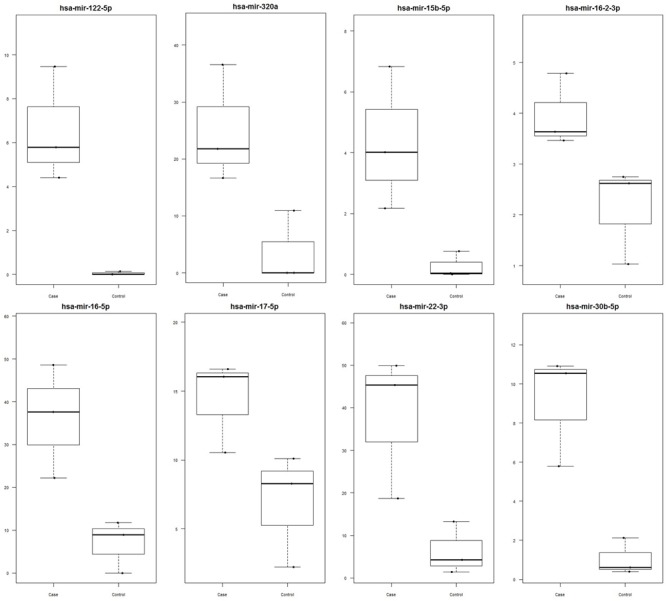
**Significantly upregulated miRNA in female versus male media as determined by Firefly^®^ Circulating miRNA Assay Immunology Panel of 68 miRNAs (ABCAM, Cambridge, MA, USA).** Data are represented as signal values for each sample. miRNAs miR-122 (*P*-value = 0.048), miR-16 (*P*-value = 0.042), miR-30b (*P*-value = 0.029), miR-320a (*P*-value = 0.042), miR-15b (*P*-value = 0.089), miR-16-2 (*P*-value = 0.061), miR-17 (*P*-value = 0.072), and miR-22 (*P*-value = 0.069) were found to be significant. Female media is represented as case and male media is represented as control.

**FIGURE 2:**
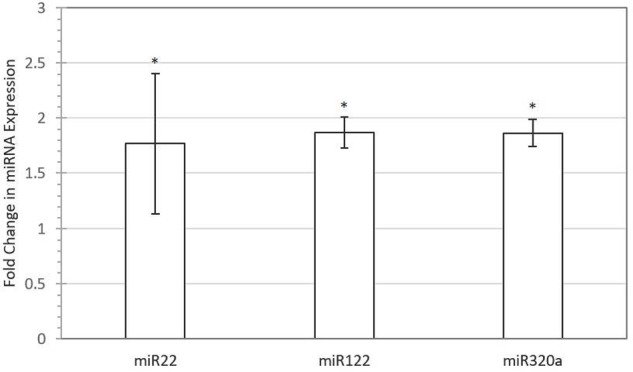
**Validation of selected miRNAs with quantitative real-time PCR (qRT-PCR).** Fold difference of expression for miR-22 (*P* < 0.05), miR-122 (*P* < 0.05), and miR-320a (*P* < 0.05) in female versus male-conditioned culture medium. Error bars represent SE for the mean fold change of the expression range. Asterisk denotes significant difference between males and females.

Out of 248 blastocysts collected through eight rounds of IVF, 158 embryos were successfully genotyped, resulting in a total of 81 males and 77 females. Thus, no significant sex bias was observed in our IVF system (*P* > 0.05). A total of 68 miRNAs were profiled using Firefly^®^ technology, of which miRNAs miR-122 (*P*-value *=* 0.048), miR-16 (*P*-value *=* 0.042), miR-30b (*P*-value *=* 0.029), miR-320a (*P*-value *=* 0.042), miR-15b (*P*-value *=* 0.089), miR-16-2 (*P*-value *=* 0.061), miR-17 (*P*-value *=* 0.072), and miR-22 (*P*-value *=* 0.069) were found to be upregulated in female-conditioned embryo media (**Figure 1**). A subset of miRNAs found to be differentially expressed in media of female embryos compared to male embryos (miR-122, miR-22, and miR-320a) was selected for validation using qRT-PCR analysis. The qRT-PCR for miR-122, miR-22, and miR-320a was performed on three biological replicates of media for embryos produced from new dams. Expression analysis revealed upregulation of all three miRNAs in the media of females, with overall fold changes of 1.77, 1.86, and 1.86 for miR-22 (*P* < 0.05), miR-122 (*P* < 0.05), and miR-320a (*P* < 0.05), respectively (**Figure 2**).

### miRNA Supplementation and Induction of Gene Expression Changes in Primary Cells

**FIGURE 3:**
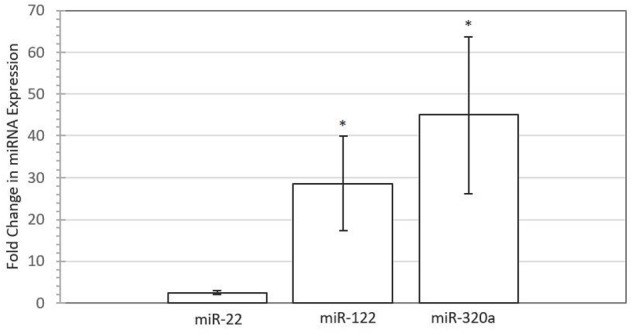
**Increased expression of miRNAs in Bovine Endometrial Epithelial Cells following supplementation with 50 nM miRNA mimic for miR-22 (*P* > 0.05), miR-122 (*P* < 0.05), and miR-320a (*P* < 0.05) compared with untreated control for four biological replicates.** Error bars represent the SE for the mean fold change of the expression range. Asterisk denotes significant difference between treated and control cells.

To test whether embryonic miRNAs are taken up by maternal endometrial cells, synthetic miRNA mimics corresponding to miR-22, miR-122, and miR-320a were supplemented to primary endometrial epithelial cells. **Figure 3** depicts the relative fold difference in expression of miRNA within BEECs following co-culture with the miRNA mimic in comparison to untreated control cells. MiR-122 (*P* < 0.05) and miR-320a (*P* < 0.05) were significantly higher in expression compared to control non-supplemented cells, with fold changes of 28.61 and 44.96, respectively. The miR-22 (*P >* 0.05) expression did not significantly differ between supplemented and control cells, though uptake was observed, with a fold change of 2.49.

**FIGURE 4:**
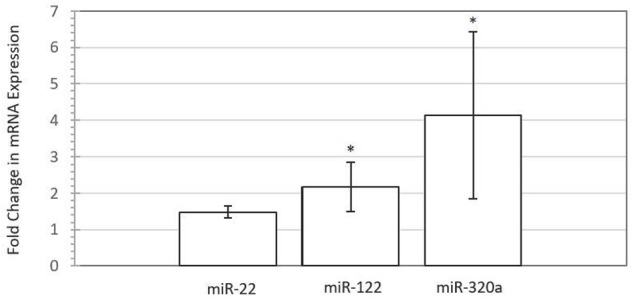
**Progesterone receptor (*PGR*) gene expression is increased following miRNA mimic (50 nM) supplementation to bovine endometrial epithelial cells for miR-22 (*P* < 0.08), miR-122 (*P* < 0.05), and miR-320a (*P* < 0.05) compared with untreated control for four biological replicates.** Error bars are represented as SE of the mean fold change for the expression range. Asterisk denotes significant difference between treated and control cells.

To assess the impact of miRNA supplementation on gene expression changes in the BEECs, we used TargetScan^3^ (Lewis et al., 2005; Agarwal et al., 2015) to search for target genes of miR-22, miR-122, and miR-320a that could have important roles in fetal-mother communication. Among several candidate genes, the progesterone receptor (*PGR*) gene was selected because it can be targeted by all three embryonic miRNAs, and is involved in the mediation of the effects of progesterone, a fundamental hormone for maintenance of pregnancy (Spencer and Bazer, 2002). **Figure 4** shows the fold difference in expression of *PGR* in endometrial cells supplemented with miRNAs miR-122, miR-22, and miR-320a compared to control cells. Interestingly, qRT-PCR analysis of a *PGR* transcript targeted by two miRNAs revealed a significant upregulation of its expression in all four biological replicates for each treatment, with fold changes of 2.17 and 4.18 for miR-122 (*P*-value *=* 0.002) and miR-320a (*P*-value *=* 0.004), respectively. A trend for upregulation of expression was also seen for cells treated with miR-22 (*P*-value = 0.077), with a fold change of 1.48. Additionally, lipofectamine-transfected cells exhibited upregulation of *PGR* gene expression for all three miRNA treatments, with fold changes of 1.51 (miR-22), 1.50 (miR-122), and 1.41 (miR-320a) when compared with a lipofectamine-only treated control (Supplementary Table 5).

## Discussion

Blastocyst-secreted miRNAs may play a remarkable role in the communication of sexually dimorphic states of conceptuses to the mother. Our findings show for the first time that male and female blastocysts differentially secrete miRNAs into culture media. Furthermore, we demonstrate that these miRNAs are taken up by maternal endometrial cells. Two miRNAs targeted *PGR* and induced upregulation of this gene, and a trend of upregulation of *PGR* was observed for the third. The observed sexually dimorphic signaling establishes a purposeful role of miRNAs in regulating the implantation process in bovine embryos.

### Sexual Dimorphism of miRNA in the Culture Media of Preimplantation Embryos

No difference in the ratio of male to female blastocysts was observed in this study, which is in agreement with other reports of *in vitro*-produced bovine embryos (Bermejo-Álvarez et al., 2010; Oliveira et al., 2016). Conversely, other studies have observed a higher proportion of male to female blastocysts (Camargo et al., 2010; Ghys et al., 2016). These differences in development reported across studies may reflect susceptibility of female conceptuses to environmental factors specific to the IVF system employed. For example, production of a higher proportion of male blastocysts was associated with a higher concentration of glucose in culture media (Kimura et al., 2008).

Epigenetic sexual dimorphism has previously been reported through observations of differential DNA methylation (Bermejo-Álvarez et al., 2008) and gene expression (Bermejo-Álvarez et al., 2008, 2010; Forde et al., 2016). Our study is the first to show sexual dimorphism in the secretion of specific miRNAs from blastocysts into the culture medium. This finding is particularly important to understanding the mechanisms of signaling within the preimplantation stage of development, as it plausibly indicates differential physiological needs of an individual embryo that can be communicated to the dam through miRNA secretion. Moreover, differential miRNA secretion could be developed into tools that serve as a method for monitoring embryonic health.

In the present study, a total of eight miRNAs were found to be upregulated in the conditioned culture media of female embryos. Indeed, patterns observed during development indicate the secreted miRNAs upregulated in female media may serve as non-invasive biomarkers for embryo growth. The observation that miR-122 decreases from hours 0 to 22 of maturation in bovine oocytes (Abd El Naby et al., 2016) and the detection of miR-320a in human embryo culture medium at the cleavage, morula, and blastocyst stages (Capalbo et al., 2016) indicate these miRNAs could serve as continuous markers of embryo developmental checkpoints. A study by Feng et al. (2015) identified that presence of miR-320 in the human follicular fluid is decreased in patients using intracytoplasmic sperm injection to conceive, and knockdown of miR-320 in mouse oocytes negatively affects developmental potential of embryos through inhibition of the Wnt signaling pathway. Additionally, miR-22 functions in human cells during apoptosis — a process which is known to be differentially regulated between male and female blastocysts (Ghys et al., 2016; Oliveira et al., 2016), suppressing tumor growth through inhibition of ATP citrate lyase (Xin et al., 2016). Further, miR-22 has been observed in media of bovine embryos which degenerate prematurely, failing to successfully form blastocysts (Kropp and Khatib, 2015). Both miR-30b (Ye et al., 2015) and miR-15 (Zhang et al., 2015) are involved in regulation of the epithelial-mesenchymal transition in cancer cells, which bears many similarities to the epithelial-mesenchymal transition important for successful implantation and gastrulation (Kalluri and Weinberg, 2009). miR-16 has been shown to inhibit angiogenesis, through targeting of vascular endothelial growth factor, serving as a potential cause of recurrent spontaneous abortions (Zhu et al., 2016). Taken together, the miRNAs found in this study may serve as non-invasive markers for the differential needs of male and female embryos.

### miRNAs as Signaling Molecules for Early Development

Significant uptake of miR-122 and miR-320a, as well as a trend of uptake for miR-22, were observed in this study. Internalization of miRNAs by endometrial cells exhibits the ability for these molecules to interact with the maternal environment and indicates their potential to function as signaling molecules. This study showed endometrial cell uptake of miRNA at half the concentration used previously by Cuman et al. (2015) on human endometrial cells, and without need of a transfection reagent. Lipofectamine 2000 was used in this study to show miRNAs (with aided transport into cells) can affect expression of *PGR*. Indeed, internalization of miRNAs, as well as increase in *PGR* was observed compared with the lipofectamine-only control.

Mechanisms through which endometrial cells take up various miRNAs have yet to be demonstrated, though the uptake of two out of three distinct miRNAs indicates passage of miRNAs into cells may be somewhat selective. It is unknown whether endometrial cells perform uptake through various modes, given that transport of extracellular miRNAs can occur through binding of the miRNA to the AGO protein, apoptotic bodies, or extracellular vesicles (microvesicles and exosomes) (Turchinovich et al., 2013). Successful transport of miRNAs could impact developmental success of the early embryo, and should thus be further interrogated.

Interestingly, the miRNAs investigated in this study have been shown to affect processes critical to the establishment of an interface between the embryo and the mother. Angiogenesis, a process necessary for embryo viability, is inhibited by miR-320 in rat myocardial microvascular endothelial cells (Wang et al., 2009). Normal rat ovarian function is linked to the roles of miR-122, which stimulates activation of the sterol response binding protein pathway, leading to LH receptor downregulation (Menon et al., 2015). The estrogen receptor ERα was shown to be downregulated by transfection with miR-22 in endometrioid carcinomas, where continuous stimulation by estrogen is considered a risk factor for tumorigenesis (Li and Yang, 2013). Thus, miRNAs found in our study have the potential to regulate cell function in the mother.

### Involvement of Progesterone Receptor in Conceptus-to-Mother Communication

Computational prediction revealed that all three miRNAs (miR-22, miR-122, and miR-320a) collectively target *PGR*, and further functional analysis showed a trend of upregulation of the *PGR* transcript following supplementation with these miRNAs individually. Interestingly, upregulation of *PGR* was correlated with the level of uptake of each miRNA into endometrial cells (**Figures 3, 4**). Most reported mechanisms for miRNA targeting of transcripts involve degradation of the transcript or inhibition of translation (Wahid et al., 2010). However, upregulation of transcripts by miRNAs has been described to occur through direct and indirect mechanisms, as well as in relation to cell state and transcript composition (Rusk, 2008; Vasudevan, 2012; Orang et al., 2014). Particularly, miR-122 has shown the ability to upregulate a transcript for hepatitis C virus by providing a scaffold for binding of essential factors to transcription, such as RNA polymerase, and enhancing binding of the 40s ribosomal subunit (Orang et al., 2014). Additional modes of upregulation include stabilization of the 5′ end of transcripts, cell cycle state-specific regulation, and protection of AU-rich regions of transcripts (Rusk, 2008; Vasudevan, 2012; Orang et al., 2014), as well as various undefined mechanisms. In the present study, the exact mechanism through which this upregulation of *PGR* occurs was not investigated. Future studies should expand on understanding the modes of upregulation which could lead to these effects on *PGR.*

Further, the substantial upregulation of the *PGR* transcript by all three miRNAs demonstrates a complex collective targeting network of miRNAs working toward regulation of a common gene. TargetScan^4^ (Lewis et al., 2005; Agarwal et al., 2015) analysis of targeting sites (data not shown) indicates all three miRNAs could bind to separate regions of the *PGR* 3′ UTR, demonstrating the possibility that several miRNAs could also simultaneously induce effects on the *PGR* transcript. This gives cause for future investigation of the potential networks conceptus-produced miRNAs may form. Interpreting these interactions may better elucidate signaling mechanisms between the conceptus and mother during preimplantation development.

The most well-studied aspect of *PGR* is its interaction with progesterone, a hormone essential to the maintenance of pregnancy and thus, upregulation of *PGR* expression could have implications for the understanding of mechanisms of cow fertility prior to and during implantation. Establishment of uterine receptivity to the conceptus has been shown to require a loss of *PGR* expression in the luminal epithelium and then later from the glandular epithelium as a result of elevated progesterone (Bazer et al., 2009). Progesterone presence in the endometrium is involved in stimulation of blastocyst growth and elongation as well as upregulation of a number of genes critical for adhesion as well as amino acid transport (Spencer et al., 2007). Studies evaluating artificial supplementation of progesterone have demonstrated an association of its administration with induction of gene expression profiles in the endometrium which are similar to expression profiles of normal endometrium interacting with more advanced conceptuses later in normal development (Forde et al., 2009). Embryos do not necessarily need to be present in the uterus at times of elevated progesterone in order to benefit from its effects (Clemente et al., 2009; O’Hara et al., 2014) and artificial elevation of progesterone early in the estrous cycle has been seen to cause decreased size of the corpus luteum, which could induce luteolysis and lead to early embryo loss (O’Hara et al., 2014). Therefore, it is possible that miRNA secretion may serve as a way for embryos to maintain a local level of control over *PGR* in order to mediate effects of progesterone on the endometrium. Although cyclic and non-cyclic cows show little divergence in endometrial transcriptomes until day 16, termed the recognition point of bovine pregnancy (Forde et al., 2011), local effects at early stages of development remain a challenge to measure and may still exist. It is unknown whether the upregulation of *PGR* in the endometrium during the preimplantation period is favorable for successful embryo development and implantation.

Our study was based on *in vitro* investigation of pooled media of blastocysts. *In vivo* models have not been employed to evaluate preimplantation embryo-secreted miRNAs. This is due partially to the challenge of developing precise techniques to measure expression changes induced by embryos within maternal tissues. For example, one study determined that transcriptomic changes occur in the epithelial cells in the oviduct as a result of embryo transfer, but individual embryo influences on the cells were undetectable and were only found to occur with the transfer of 50 embryos (Maillo et al., 2015). Further investigation of these secreted miRNAs should also lead to a better understanding of the differences between individual blastocysts in order to uncover the biology behind this secretion and to develop ways to monitor individual conceptuses throughout development.

Although this study was limited to a panel of miRNAs chosen specifically for their impacts on immunity as opposed to a high-throughput sequencing approach, the results provide a basis for identifying larger-scale dimorphisms of secreted miRNAs. A comprehensive understanding of the exact roles for sexually dimorphic miRNA production has not yet been attained, but future research should focus on understanding larger-scale interactions and collective targeting of subsets of miRNAs on mRNA transcripts in order to better elucidate the roles these molecules may play in maternal cells and to accommodate optimal development of blastocysts relative to their sex.

## Conclusion

The current study demonstrates the potential for blastocysts of differing sex to produce dimorphic signals in the form of miRNAs, which can collectively impose changes in transcripts of maternal cells. Endometrial cell internalization of miRNAs, and subsequent upregulation of *PGR* is a remarkable display of the potential for miRNAs to function as signaling molecules during preimplantation development and allows for a new perspective on understanding the preparation of maternal cells for interaction with blastocysts, which already contain inherent differences due to their sex. Male and female embryo dimorphism in miRNA production allows for future discovery of invaluable biomarkers of embryo signaling, and understanding the potential roles of miRNAs in preimplantation development blastocyst-to-mother communication.

## Author Contributions

NG carried out the experiments, data analysis and drafting of the manuscript. JK participated in the *in vitro* production of embryos and editing the manuscript. HK conceived the study and participated in the design of experiments and drafting of the manuscript.

## Conflict of Interest Statement

The authors declare that the research was conducted in the absence of any commercial or financial relationships that could be construed as a potential conflict of interest.

## Footnotes

1 www.miRBase.org

2 www.targetscan.org

3 www.targetscan.org

4 www.targetscan.org
