# Supplementary material for: Sexual Dimorphism of miRNAs Secreted by Bovine In vitro-produced Embryos
Source: Front Genet. 2017 Apr 4;8:39. doi: 10.3389/fgene.2017.00039 (PMC5378762; doi:10.3389/fgene.2017.00039)
Supplement: Supplementary file 1 [file Table_1.DOC]

**Supplementary Table 1.** PCR primer sequences used for sex determination of embryos

|  | **Gene** | **Primer Sequence**  **(5’–3’)** | **Amplicon Size**  **(bp)** |  | |
| --- | --- | --- | --- | --- | --- |
|  | zfx/zfy | Forward: ATAATCACATGGAGAGCCACAAGCT | 445/447 | |  |
|  |  | Reverse: GCACTTCTTTGGTATCTGAGAAAGT |  |  |  |
|  | Zfx | Forward: GACAGCTGAACAAGTGTTACTG | 247 | |  |
|  |  | Reverse: AATGTCACACTTGAATCGCATC |  |  |  |
|  | Zfy | Forward: GAAGGCCTTCGAATGTGATAAC | 167 | |  |
|  |  | Reverse: CTGACAAAAGGTGGCGATTTCA |  |  |  |

***** Primer sequences zfx/zfy were obtained from Aasen and Medrano (1990) and Zfx and Zfy obtained from Kirkpatrick and Monson (1993).
