# Supplementary material for: Sexual Dimorphism of miRNAs Secreted by Bovine In vitro-produced Embryos
Source: Front Genet. 2017 Apr 4;8:39. doi: 10.3389/fgene.2017.00039 (PMC5378762; doi:10.3389/fgene.2017.00039)
Supplement: Supplementary file 2 [file Table_2.DOC]

**Supplementary Table 2.** Summary of embryo generation and genotyping

|  | **Purpose of Generation** | **Total Generated** | **Successfully Genotyped** | **Male** | **Female** |
| --- | --- | --- | --- | --- | --- |
|  | miRNA profiling | 102 | 57 | 30 | 27 |
|  | Validation of miRNA profiling | 146 | 101 | 51 | 50 |
