# Supplementary material for: Sexual Dimorphism of miRNAs Secreted by Bovine In vitro-produced Embryos
Source: Front Genet. 2017 Apr 4;8:39. doi: 10.3389/fgene.2017.00039 (PMC5378762; doi:10.3389/fgene.2017.00039)
Supplement: Supplementary file 3 [file Table_3.DOC]

**Supplementary Table 3.** Mature miRNA sequences

|  | **miRNA** | **Sequence**  **(5’–3’)** | |  | |  | |
| --- | --- | --- | --- | --- | --- | --- | --- |
|  | miR-22 | AAGCUGCCAGUUGAAGAACUGU | |  |  | | |
|  | miR-122 | UGGAGUGUGACAAUGGUGUUUG |  | | | |  |
|  | miR-320a | AAAAGCUGGGUUGAGAGGGCGA | |  |  | | |
