# Supplementary material for: Sexual Dimorphism of miRNAs Secreted by Bovine In vitro-produced Embryos
Source: Front Genet. 2017 Apr 4;8:39. doi: 10.3389/fgene.2017.00039 (PMC5378762; doi:10.3389/fgene.2017.00039)
Supplement: Supplementary file 4 [file Table_4.DOC]

**Supplementary Table 4.** qRT-PCR primer sequences

|  | **Gene** | **Primer Sequence**  **(5’–3’)** | **Amplicon Size**  **(bp)** | |  |
| --- | --- | --- | --- | --- | --- |
|  | *PGR* | Forward: GTCCCTAGCTCACAGCGTTT | 111 |  | |
|  |  | Reverse: TGCCCGGGACTGGATAAATG |  |  | |
|  | Beta-Actin | Forward: AGGCCAACCGTGAGAAGATGAC | 100 |  | |
|  |  | Reverse: CCAGAGGCATACAGGGACAGC |  |  | |
