# Supplementary material for: Sexual Dimorphism of miRNAs Secreted by Bovine In vitro-produced Embryos
Source: Front Genet. 2017 Apr 4;8:39. doi: 10.3389/fgene.2017.00039 (PMC5378762; doi:10.3389/fgene.2017.00039)
Supplement: Supplementary file 5 [file Table_5.DOC]

**Supplementary Table 5.** *PGR* gene expression results for lipofectamine-treated cells

|  |  | **miR-22** | **miR-122** | **miR-320a** |  |
| --- | --- | --- | --- | --- | --- |
|  | Fold change in expression of *PGR* | 1.51 | 1.50 | 1.41 |  |

*Gene expression changes are relative to a lipofectamine-only treated control.
